# Automated segmentation and volume prediction in pediatric Wilms’ tumor CT using nnu-net

**DOI:** 10.1186/s12887-024-04775-2

**Published:** 2024-05-09

**Authors:** Weikang Li, Yiran Sun, Guoxun Zhang, Qing Yang, Bo Wang, Xiaohui Ma, Hongxi Zhang

**Affiliations:** 1 Department of Radiology, The Children’s Hospital, Zhejiang University School of Medicine, National Clinical Research Center for Child Health, No. 3333, Binshneg Rd, Hangzhou, China; 2 Wenzhou Medical University, Wenzhou, China

**Keywords:** CT, Wilms’ Tumor, Deep learning

## Abstract

**Background:**

Radiologic volumetric evaluation of Wilms’ tumor (WT) is an important indicator to guide treatment decisions. However, due to the heterogeneity of the tumors, radiologists have main-guard differences in diagnosis that can lead to misdiagnosis and poor treatment. The aim of this study was to explore whether CT-based outlining of WT foci can be automated using deep learning.

**Methods:**

We included CT intravenous phase images of 105 patients with WT and double-blind outlining of lesions by two radiologists. Then, we trained an automatic segmentation model using nnUnet. The Dice similarity coefficient (DSC) and 95th percentile Hausdorff distance (HD_95_) were used to assess the performance. Next, we optimized the automatic segmentation results based on the ratio of the three-dimensional diameter of the lesion to improve the performance of volumetric assessment.

**Results:**

The DSC and HD_95_ was 0.83 ± 0.22 and 10.50 ± 8.98 *mm*. The absolute difference and percentage difference in tumor size was 72.27 ± 134.84 *cm*^*3*^ and 21.08% ± 30.46%. After optimization according to our method, it decreased to 40.22 ± 96.06 *cm*^*3*^ and 10.16% ± 9.70%.

**Conclusion:**

We introduce a novel method that enhances the accuracy of predicting WT volume by integrating AI automated outlining and 3D tumor diameters. This approach surpasses the accuracy of using AI outcomes alone and has the potential to enhance the clinical evaluation of pediatric patients with WT. By intertwining AI outcomes with clinical data, this method becomes more interpretive and offers promising applications beyond Wilms tumor, extending to other pediatric diseases.

## Introduction

Wilms’ tumor (WT) is one of the most common solid tumors in infants and children, ranking second in primary abdominal malignancies in children after neuroblastoma [1]. The diagnosis of WT relies heavily on imaging, such as abdominal plain film, excretory urography, abdominal ultrasound, abdominal CT or MRI [2]. Among these, plain abdominal CT scans and enhancement scans are the most important examinations, with a diagnostic accuracy of over 95% [3]. Treatment for WT typically involves a combination of surgery and chemotherapy, resulting in a survival rate of up to 90% [3]. The choice of treatment primarily depends on clinical staging [4].

Currently, puncture biopsy is still the main tool for the clinical diagnosis of WT. However, in children, puncture biopsy not only increases injury but also poses risks. The North American Children’s Oncology Research Collaborative (COG) believes that preoperative application of fine-needle aspiration biopsy, core-needle biopsy, or open biopsy may lead to increased recurrence and mortality rates. This would result in the upgradation of WT to Stage III, requiring the addition of radiation therapy, which can further damage the child’s organism [5]. On the other hand, the European Society for International Pediatric Oncology (SIOP), another authoritative organization for WT research, has ruled that preoperative open wedge biopsy artificially ruptures the tumor envelope, upgrading WT to stage III. However, fine-needle aspiration biopsy or core-needle biopsy is not a criterion for upgrading to stage III [6].

Tumor volume measurement is crucial for the treatment of nephroblastoma. It is considered an indicator of response to therapy and risk stratification, as suggested by the SIOP UMBRELLA guidelines [2]. According to these guidelines, patients with a unilateral tumor volume of less than 300 ml at the time of diagnosis and without susceptibility tumor syndrome exclusively undergo renal unit-sparing surgery (NSS) [7, 8]. Furthermore, tumor volume has been found to have predictive value in determining patient prognosis [9, 10]. However, the current method for tumor volume measurement relies on manual outlining of the lesion by radiologists, making it a labor-intensive and experience-dependent process.

Deep learning, a branch of artificial intelligence, utilizes neural networks for learning. By applying a specific network structure, accurate manual results can be achieved. However, extensive training can automate this process [11]. Many scholars have conducted research on deep learning, recognizing its potential for automatically segmenting lesions and saving time while minimizing subjective errors. The objective of this study is to explore the application of deep learning for the segmentation of WT lesions and evaluate the feasibility of automated segmentation for imaging analysis [12–14].

## Materials and methods

### DATA

Retrospective analysis was conducted on data from 106 patients with WT who were hospitalized at Children’s Hospital of Zhejiang University between October 2014 and October 2021. The inclusion criteria were as follows: (i) pathologically verified WT; (ii) abdominal enhancement CT prior to treatment; and (iii) CT-enhanced images including the portal phase.

A total of 105 patients were enrolled in the study after excluding those with low-resolution images or motion artifacts. Among the 105 patients included in the analysis, 51 were male (49%), and 54 were female (51%). The median age at 24 months ranged from 1 to 123 months (Table 1). To ensure unbiased results, all patients were randomly divided into two groups: the training group (*n* = 75) and the test group (*n* = 30).

**Table 1:** Baseline characteristics (*n* = 105). Numbers are count unless otherwise specified

DATASET	Sex		Age (month)	
	Male	Female	Median	Mean
*Train*	36	39	21	26.57
*Test*	15	15	36.5	42.79
*Total*	51	54	24	31.21

According to the COG staging guidelines, tumors were classified based on whether the tumor was confined to the kidney or not. Based on this categorization, we classified patients into stage I and > I.

### Image acquisition

All CT examinations were randomized using a 16-row CT (Siemens Somatom Emotion 16, Germany) and 64-row CT (GE Optima CT660, Japan) were performed (Table 2). Various scanning parameters lead to differences in CT image resolution. We believe that enhancing the dataset’s complexity will help address the constraints of depending solely on a single-center data source.

**Table 2:** CT imaging parameters

Parameters	Siemens CT	GE CT
Tube voltage	110 *kV*	120 *kV*
Tube current	75 *mA*	80 *mA*
Layer thickness	1.5 *mm*	0.625 *mm*
FOV	350 *mm*$\times$ 350 *mm*	350 *mm*$\times$ 350 *mm*
Matrix	512 $\times$ 512	512 $\times$ 512

After CT scanning, nonionic iodinated contrast agent was injected intravenously using a high-pressure syringe at a dose of 1.5 *ml/kg* body weight at a flow rate of 1.5-2 *ml/sec*, with a delay of 50 s for acquiring intravenous phase images. The study was approved by the hospital ethics committee.

### Image processing

The whole process involved outlining the regions of interests (ROIs) for all renal masses in WT patients using portal intravenous CT images. In collaboration with two diagnostic radiologists with more than 10 years of clinical experience, the 3D Slicer platform was employed for this purpose. To analyze the interobserver reproducibility, the two diagnostic imaging physicians blindly segmented 30 randomly selected tumor ROIs. To assess intraobserver reproducibility, these 30 tumor ROIs were repeatedly outlined over a 2-week period following the same procedure.

Tumor size was calculated by multiplying the number of voxels in the tumor segmentation by the voxel size in *cm*^*3*^.

### Deep learning

**Fig. 1:**
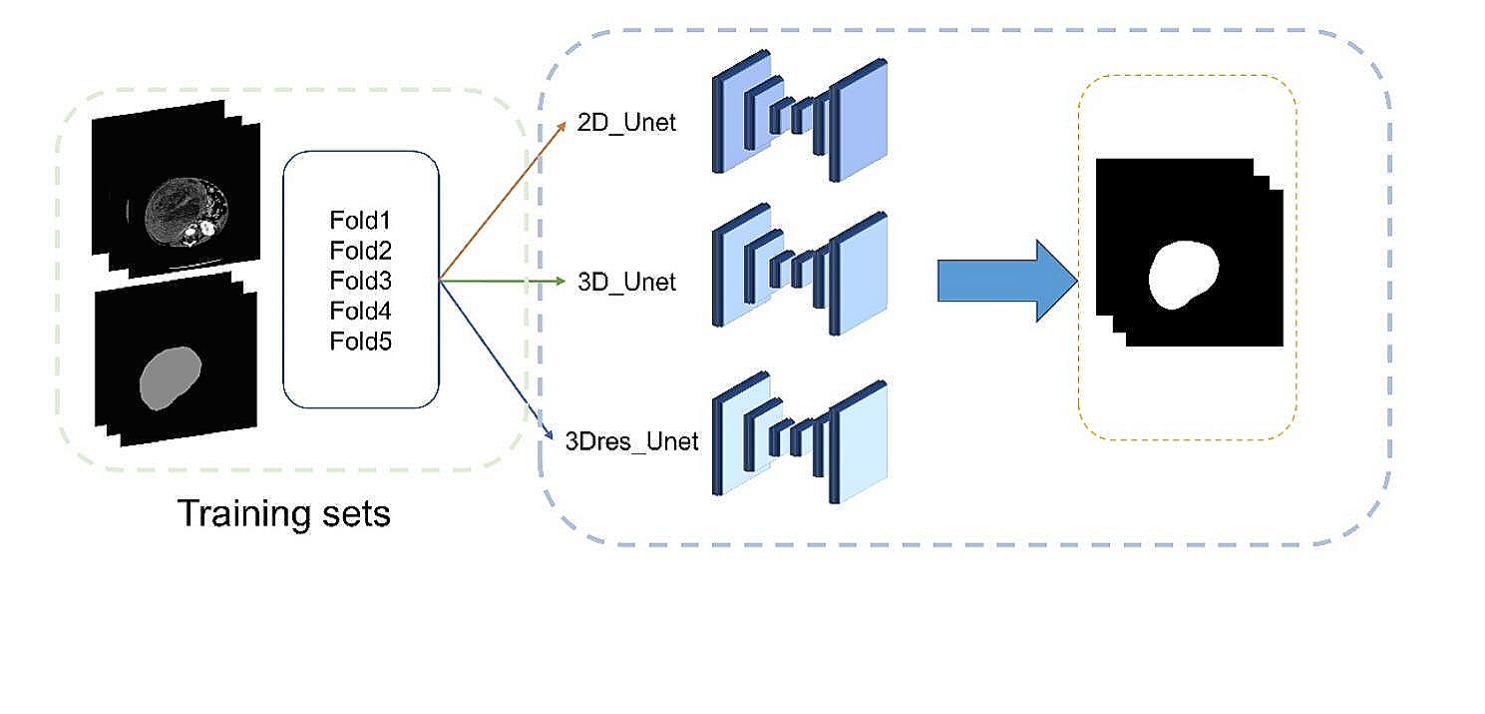
Overview of the deep learning workflow. The image in the figure is a 2D schematic representation of the 3D volume

The deep learning network nnU-Net, a U-net-based adaptive segmentation framework, was employed for automatic tumor segmentation [15]. The source code for nnU-Net is publicly available on GitHub (https://github.com/MIC-DKFZ/nnunet). Three models, namely, 2D Unet, 3D Unet, and 3Dres Unet (U-Net Cascade), were trained using the training set data. To fully utilize the patient data, a 5-fold cross-validation strategy was applied throughout the training process. After training, the outputs of the three models were combined in pairs to further fuse the results. Subsequently, the best model from the selection was validated on the test set of images. The results were then compared and analyzed with the manual segmentation results. An overview of the deep learning workflow is shown in Fig. 1.

We trained all neural networks for 1000 iterations. During training, the 2D and 3D networks were sampled at 512 × 512 and 80 × 192 × 160, with batch sizes of 12 and 2, respectively. The models were implemented using Python (3.9) and PyTorch (2.1).

### Statistical analysis

The Dice similarity coefficient (DSC) would be utilized to evaluate both inter- and intra-observer consistency. Reproducibility was deemed satisfactory if DSC > 0.9. To evaluate the performance of automated tumor volume measurements based on deep learning, the quality of automated segmentation was assessed using DSC and the 95th percentile Hausdorff distance (HD_95_). Furthermore, intergroup correlation coefficients were employed to evaluate the agreement between automatic and manual segmentation. The statistical procedure was performed using Python 3.9, and *P* < 0.05 was considered significantly different.

## Results

### Distribution of tumors

The distributions of tumor sizes in the total dataset and the training set are relatively close to each other, with median and mean values not differing much and consistent ranges. Both the total dataset and the training set have slightly higher median and mean values compared to the test set. In the total dataset, the ratio of the number of stage I tumors to nonstage I tumors was 0.78. This ratio was similar in the training set, while in the test set, it was lower at 0.67 (Table 3).

**Table 3:** Tumors Information

DATASET	Tumor staging		Tumor Size(cm^3^)	
	Stage I	Stage > I	Median	Mean
***Train(*** ***n*** *** = 75)***	34	41	324.9	409.9
***Test(*** ***n*** *** = 30)***	12	18	154.8	298.2
***Total(*** ***n*** *** = 105)***	46	59	315.5	378.0

### Deep learning-based segmentation

Based on the three networks, we performed five-fold cross-validation in the training set and selected the model with the highest DSC for each network (Table 4). These models were then combined, resulting in six segmentation models. Among the six models, 3D-3Dres, the combined model, produced the best segmentation effect. It achieved a median DSC of 0.9489, a mean of 0.8976, a median HD95 of 5.39 *mm*, and a mean of 11.29 *mm*. Hence, the 3D-3Dres model was selected as the best model for testing.

**Table 4:** Deep learning training results

TRAIN	DSC		HD 95(mm)	
	Median	Mean	Median	Mean
***2D_Unet***	0.9409	0.8878	6.5574	16.7306
***3D_Unet***	0.9488	0.8913	5.7446	11.8477
***3Dres_Unet***	0.9443	0.8941	5.7446	12.4469
***2D-3D_Unet***	0.9468	0.8937	5.3852	12.9013
***2D-3Dres_Unet***	0.9463	0.8971	5.3852	11.5593
***3D-3Dres_Unet***	0.9489	0.8976	5.3852	11.2877

The results of the statistical analysis comparing the tumor lesions predicted by the BEST model with manual segmentation in the test set showed that the entire test set had a median DSC value of 0.9296, with a mean value of 0.8543 and a range of 0.2927–0.9715. Three cases had a DSC value less than 0.6. Moreover, the median HD_95_ value was 8.093 *mm*, with a mean value of 10.50 *mm* and a range of 2.449–39.87 *mm*. Two cases had values exceeding 30 *mm* (Table 5). Additionally, when assessing the autosegmented lesions, more consistent results were obtained for both stage I and nonstage I tumors. The median DSC value was above 0.9, with a mean value above 0.85. The median HD_95_ value was approximately 8 *mm*, with a mean value of approximately 10.5 *mm*. None of these values showed statistical significance.

**Table 5:** Deep learning testing results

TEST	DSC		HD_95(_mm)	
	Median	Mean	Median	Mean
***Stage I (*** ***n*** *** = 12)***	0.9300	0.8548	8.0623	10.1052
***> Stage I (*** ***n*** *** = 18)***	0.9158	0.8540	8.3632	10.7680
***Total (*** ***n*** *** = 30)***	0.9296	0.8543	8.0931	10.5029

**Fig. 2:**
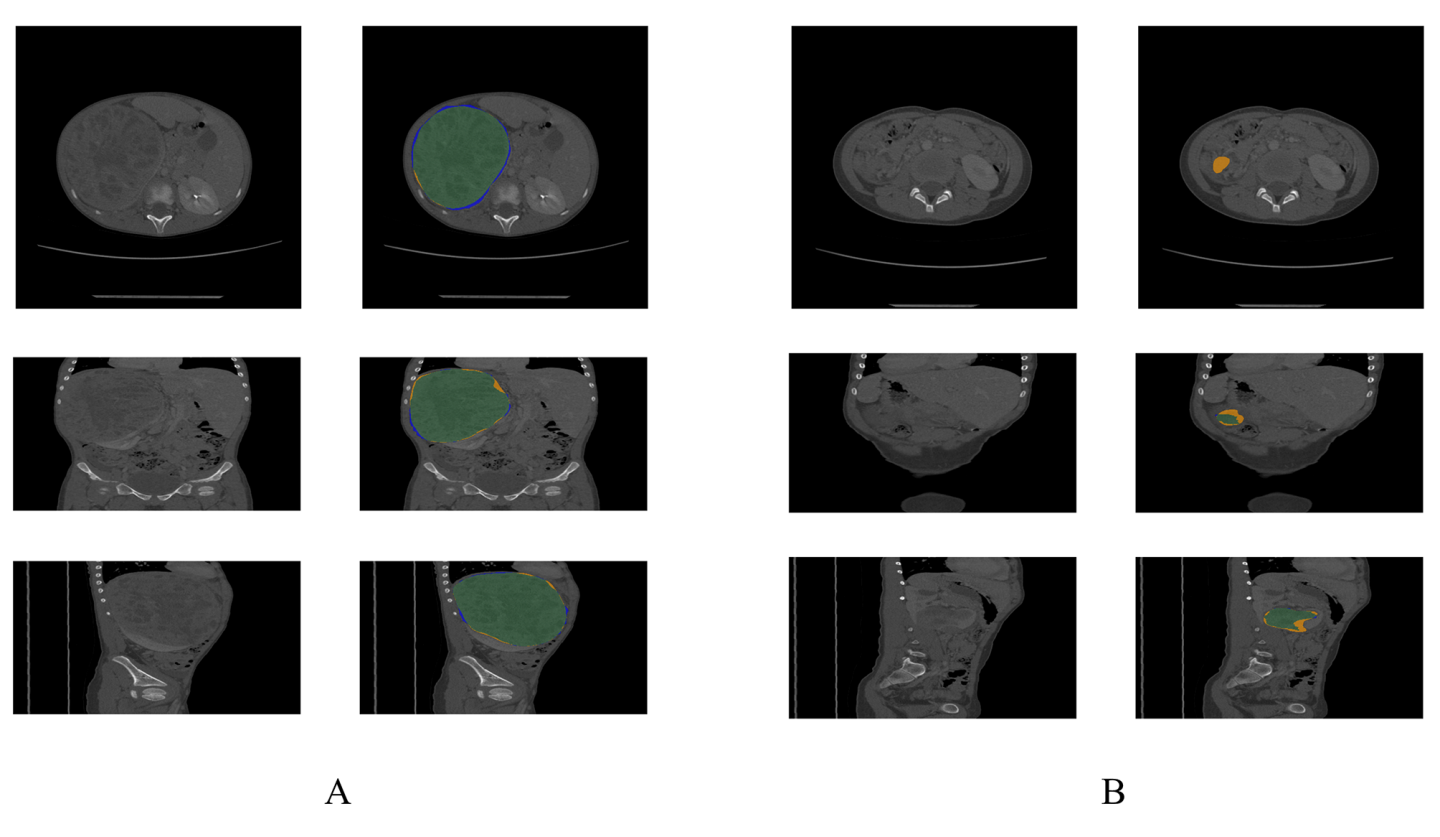
shows tumor lesions with the highest and lowest DSC scores. It is evident that in tumors with a high DSC score, the automatically segmented lesions closely match the manually segmented lesions. In contrast, tumors with a low DSC score exhibit larger automatically segmented foci than manually segmented foci. Additionally, these foci are identified in regions that physicians would usually classify as lesion-free

Figure 2. Examples of automated prediction of tumor lesions. A: CT image of a patient with DSC of 0.9715. B: CT image of a patient with DSC of 0.2927. In each set of images, the left column is the original image and the right column is the lesion image. Each row displays horizontal, coronal and sagittal images in order from top to bottom. In the lesion image, the green area is the overlapping part of manual segmentation and automatic segmentation, the blue area is the manual segmentation only, and the yellow area is the automatic segmentation only.

### Tumor size

Based on the manual segmentation size of tumors (0-300 *cm*^*3*^, 300–500 *cm*^*3*^, and > 500 *cm*^*3*^), there was a significant increase in the difference between automatically segmented and manually segmented volumes. Particularly, the difference was highest in the 300–500 *cm*^*3*^ tumor size group, surpassing the other groups (Table 6). For tumor volumes below 300 *cm*^*3*^, both the absolute and percentage differences were smaller compared to the total group. For tumor volumes exceeding 500 *cm*^*3*^, the absolute difference was second highest after the 300–500 *cm*^*3*^ group.

**Table 6:** Absolute and percentage differences in tumor size between automatic and manual segmentation

Tumor Size(cm^3^)	Absolute Difference(cm^3^)		Percentage Difference	
	DL	Pre	DL	Pre
***< 300 (*** *n* *** = 17)***	15.96	6.36	21.89%	9.27%
***300–500 I (*** ***n*** *** = 8)***	301.85	183.82	34.84%	20.42%
***> 500(*** ***n*** *** = 5)***	48.43	22.41	10.76%	5.64%
***Total (*** ***n*** *** = 30)***	72.27	40.22	21.08%	10.16%

^a^ The values in the table are averages

^b^ DL and Pre represent the automatic tumor segmentation volume and optimized prediction volume, respectively

**Fig. 3:**
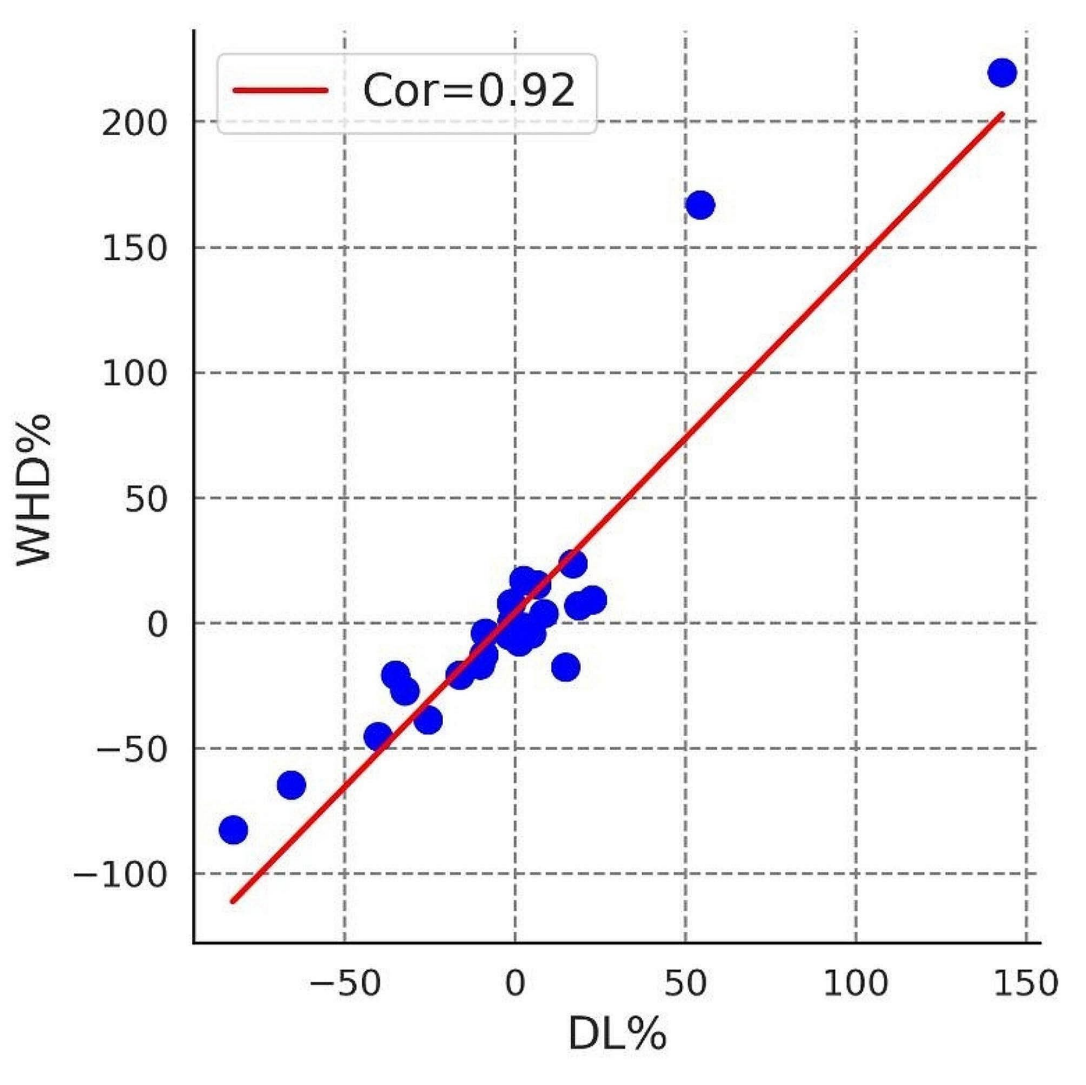
Linear correlation plot of the percentage difference in volume and three-dimensional maximum diameter product between the lesions obtained from automatic segmentation and manual segmentation. DL% is the percentage difference in volume, and WHD% is the percentage difference in three-dimensional maximum diameter

When calculating the tumor volume, we found a strong correlation between the product of the 3D diameter ratio and the tumor size for automatically segmented and manually segmented lesions (Fig. 3).

**Fig. 4:**
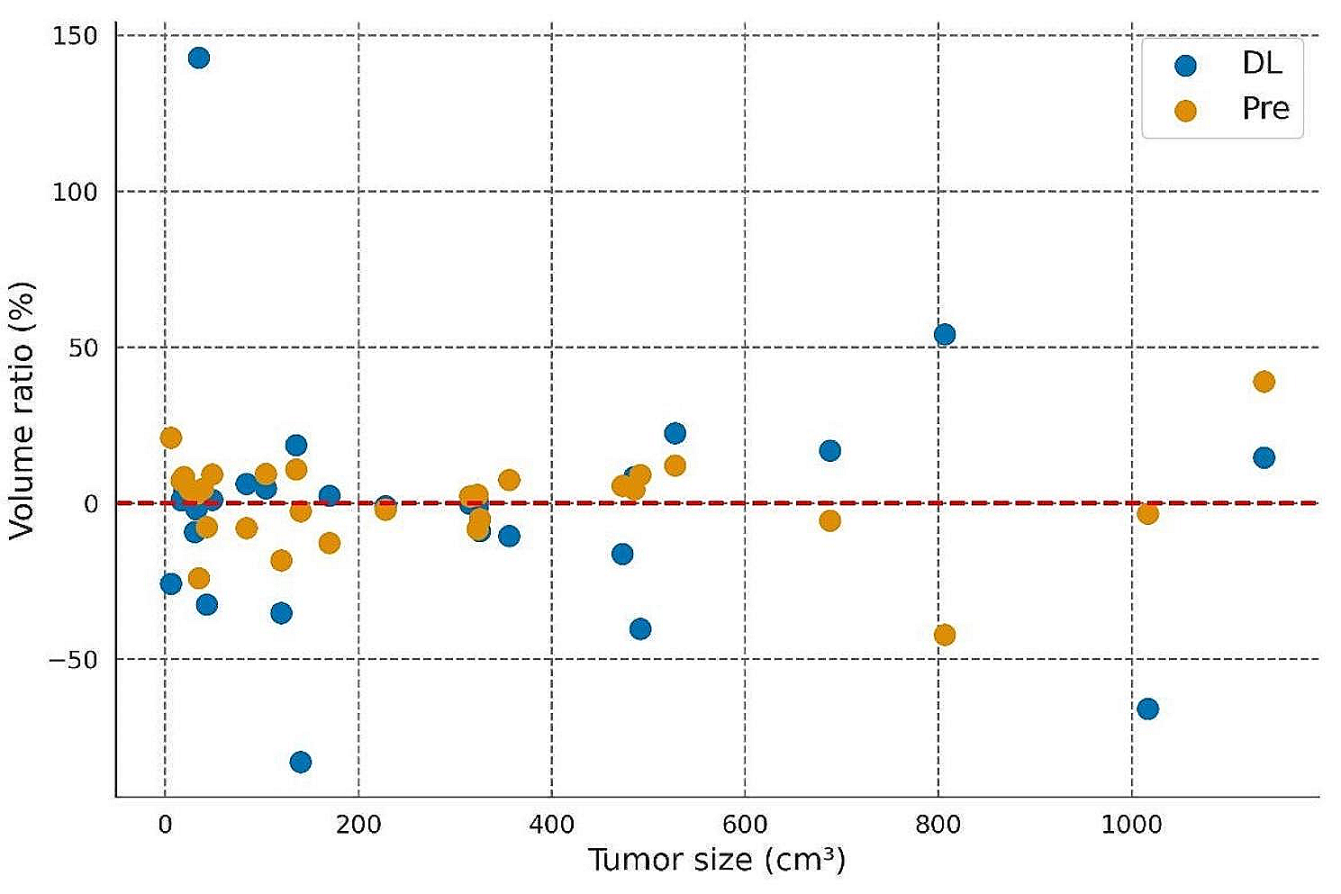
Two deep learning-based vs. manual-based tumor volumes. The x-axis is the artificial tumor volume and the y-axis is the percentage difference in predicted volume. DL and Pre represent the automatic tumor segmentation volume and optimized prediction volume, respectively. The red line corresponds to no difference with the manually determined reference volume. Points above the red line indicated that the model underestimates tumor volume, and points below the red line indicated that it overestimates tumor volume

Specifically, for tumor sizes between 300 and 500 *cm*^*3*^, the mean difference decreased by 118.03 *cm*^*3*^ (39.10%), and the mean percentage difference decreased by 14.42% (Table 6). Figure 4 illustrates the percentage error distribution of the two volume measurement methods.

**Fig. 5:**
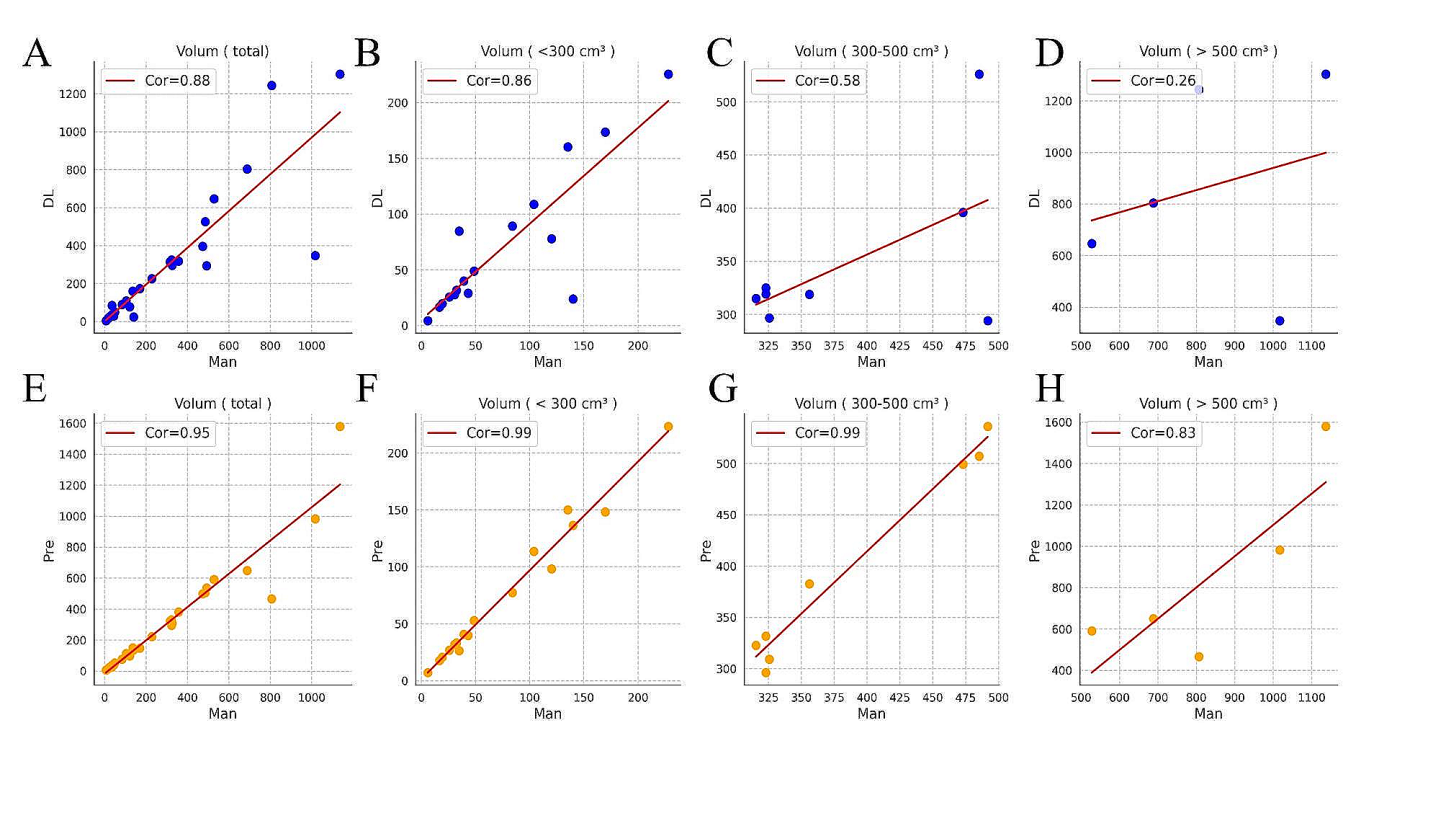
Pearson’s correlation coefficients between manual segmentation and automated segmentation and 3D predicted tumor volumes, respectively. (**A**-**D**) Scatterplots representing the automated segmentation. (**E**-**H**) Scatterplots representing the 3D predicted. Man, manual segmentation volumes; DL, automated segmentation volumes; Pre, 3D predicted tumor volumes

The predicted volume based on the 3D diameter ratio was more accurate and in better agreement with manual segmentation than the direct calculation of volume after automatic segmentation. There was no significant difference between the volume of both methods and the volume of manual segmentation (P_DL_=0.93, P_Pre_=0.95). The overall consistency of automatically segmented tumor size was strong. However, this consistency weakened significantly when the tumor volume exceeded 300 *cm*^3^ (Fig. 5). Conversely, the predicted volume exhibited superb consistency (ICC > 0.95), except for the > 500 cm3 group (ICC = 0.83), demonstrating the effectiveness of the prediction method.

## Discussion

The results of this study demonstrate that deep learning can partially replace manual tumor lesion segmentation. Moreover, when combined with manual measurement of the maximum diameter of the three dimensions, deep learning can provide more accurate predictions of tumor size. This further supports the feasibility of automated lesion segmentation in tumor radiomics studies.

In terms of the clinical information, the clinical details of the training and test sets in our data are largely similar to those of the total data. However, it is worth noting that patients in the test set were older. For tumor lesion size, the distribution of the training set closely resembles that of the total dataset, whereas the lesion size in the test set is slightly smaller. The smaller lesion size in the test set might have affected the model’s validation [16, 17]. Despite this, our trained automatic segmentation model exhibited relatively good performance in the test set, with a median DSC value of 0.93 and a mean value of 0.85. Nevertheless, the model did not adequately identify lesion edges in certain tumors. The lowest DSC value in the test set was 0.29, indicating oversegmentation of the tumor lesion for this particular patient (Fig. 2). This outcome could be attributed to the low frequency of small lesions in the test set, resulting in a decrease in the model’s accuracy in identifying such lesions [17].

Based on the variations in tumor size observed among patients, it appears that tumor size plays a crucial role in model performance. Our analysis reveals a range of tumor sizes, with the smallest tumor measuring 5.9 *cm*^*3*^ and the largest tumor measuring 1136.8 *cm*^*3*^, indicating a significant difference of 1130.9 *cm*^*3*^ (19,268%). Figures 2 and 4a illustrate the distribution of manually segmented lesion sizes, which appears relatively discrete, while the automatically segmented lesion sizes demonstrate a more centralized distribution. We attribute this difference to the mathematical nature of the deep learning algorithm, which favors continuous data types. Additionally, the limited data available and the incomplete understanding of the actual complex distribution might contribute to this disparity. Our evaluation of tumor size grouping shows that the model performs significantly better in segmenting small tumors (less than 300 *cm*^*3*^) compared to other tumor sizes. However, the worst performance is observed in segmenting average-sized tumors (300–500 *cm*^*3*^). We hypothesize that the correlation between tumor size and the degree of deterioration plays a role in this disparity. As tumor size increases, it may invade surrounding tissues, and the extent of invasion varies across patients, leading to the model’s inadequate understanding of this feature. The slightly suboptimal segmentation of large tumors (> 500 *cm*^*3*^) may be due to the model’s relative proficiency in segmenting large tumors, given the limited presence of only five large tumors in the test set, which may have occurred by chance. To validate these findings further, expanding the sample size is necessary.

Regarding the tumor stage, we currently only focus on stage I patients because this will directly affect the choice of subsequent surgical strategies. The small amount of data is one of the reasons why we did not make further distinctions for patients with other stages. Additionally, we collected preoperative examinations from patients with low tumor deterioration and fewer patients with high-grade tumors. Our data confirm that tumor staging I or not had no significant effect on manual and automated segmentation. It is worth noting that the number of different pathological subtypes of WT in the dataset can affect the model performance of deep learning segmentation, as shown by Buser, Myrthe A D et al. [18]. However, it is important to clarify that the task of tumor staging classification belongs to our previous work [19], and the focus of this study was on lesion segmentation independent of tumor staging.

In clinical practice, the routine practice for describing the size of a tumor is to use the maximum diameter in three directions [20–23]. However, it has been found that this method underestimates the actual volume of tumors. Müller et al. conducted a study on Wilms tumors and discovered that the volume was underestimated by an average of 22% when compared to measurements made by human experts using ellipsoid-based measurements [24]. Similarly, a research study by Buser et al. found that ellipsoid-based measurements underestimated volumetric measurements based on manual segmentation by an average of 10% [18]. Although manual segmentation is highly accurate, it is a time-consuming process and heavily relies on the experience of the radiologist [24, 25].

In our study, automated segmentation combined with manual outlining of the 3D maximum diameter could more accurately predict the true size of the tumor. The maximum diameter indirectly represents tumor size [26]. This method exhibits less error and more stability compared to automatic segmentation of lesions. However, due to the lack of samples, the model is less effective in segmenting large tumors and does not agree well with manually segmented tumors. Nonetheless, the prediction method effectively compensates for this limitation, as the predicted tumor size is highly consistent with manual segmentation.

This study has some limitations that need to be acknowledged. First, our sample size was small, as it only included WT patients from our institution between 2014 and 2021. Nonetheless, when compared to the study conducted by Buser, Myrthe A D et al., our sample size still stands out [24]. Second, we were unable to determine the specific pathological staging and tumor type of WT patients at our institution, which necessitates further investigation to exclude any potential pathological differences. Third, although the prediction method has improved the application of automated segmentation, its effectiveness for clinical decision-making has yet to be fully explored. Fourth, while we collected data from two different devices and had two physicians perform lesion segmentation, it should be noted that all the patients were from the same institution. Consequently, we plan to conduct a large-scale multidisciplinary multidisease cohort study to validate our findings.

## Conclusions

Our study confirms the efficacy of deep learning in automatically outlining WT lesions. Additionally, we introduce a novel approach for predicting WT volume by integrating tumor three-bit diameter measurements with AI-generated outcomes. This advanced method not only enhances efficiency and precision but also demonstrates greater reliability compared to using AI alone for volume prediction. The implementation of this technique has the potential to enhance the clinical evaluation of pediatric patients with WT and may even impact treatment strategies for individual cases. Furthermore, the application of this combined 3D diameter approach for volume prediction holds promise for potential adaptations to other types of pediatric tumors and could serve as a benchmark for future research endeavors.

## Data Availability

The datasets analyzed during the current study are not publicly available due to patient privacy. Upon reasonable request to the corresponding author the raw data can be made available, however the imaging itself will not be shared.

## Abbreviations

WT: Wilms’ tumor
DSC: Dice similarity coefficient
HD95: The 95th percentile Hausdorff distance
COG: The North American Children’s Oncology Research Collaborative
SIOP: The European Society for International Pediatric Oncology
NSS: Unit-sparing surgery
ROIs: The regions of interests

## References

[1] Spreafico F, Bellani FF (2006). Wilms’ tumor: past, present and (possibly) future. Expert Rev Anticancer Ther.

[2] SIOP Renal Tumour Study Group (2017). Paediatric renal tumours: perspectives from the SIOP-RTSG. Nat Rev Urol.

[3] Malogolowkin M, Cotton CA, Green DM, Breslow NE, Perlman E, Miser J, Ritchey ML, Thomas PR, Grundy PE, D’Angio GJ, Beckwith JB, Shamberger RC, Haase GM, Donaldson M, Weetman R, Coppes MJ, Shearer P, Coccia P, Kletzel M, Macklis R, Tomlinson G, Huff V, Newbury R, Weeks D, National Wilms Tumor Study Group (2008). Treatment of Wilms tumor relapsing after initial treatment with vincristine, actinomycin D, and doxorubicin. A report from the National Wilms Tumor Study Group. Pediatr Blood Cancer.

[4] Roy P, van Peer SE, de Witte MM, Tytgat GAM, Karim-Kos HE, van Grotel M, van de Ven CP, Mavinkurve-Groothuis AMC, Merks JHM, Kuiper RP, Hol JA, Janssens GOR, de Krijger RR, Jongmans MCJ, Drost J, van der Steeg AFW, Littooij AS, Wijnen MHWA, van Tinteren H, van den Heuvel-Eibrink MM (2022). Characteristics and outcome of children with renal tumors in the Netherlands: the first five-year’s experience of national centralization. PLoS ONE.

[5] Dome JS, Fernandez CV, Mullen EA, Kalapurakal JA, Geller JI, Huff V, Gratias EJ, Dix DB, Ehrlich PF, Khanna G, Malogolowkin MH, Anderson JR, Naranjo A, Perlman EJ, COG Renal Tumors Committee (2013). Children’s Oncology Group’s 2013 blueprint for research: renal tumors. Pediatr Blood Cancer.

[6] PDQ Pediatric Treatment Editorial Board. Wilms Tumor and Other Childhood Kidney Tumors Treatment (PDQ®): Patient Version. 2021 Aug 18. In: PDQ Cancer Information Summaries [Internet]. Bethesda (MD): National Cancer Institute (US); 2002&#8211. 26389390

[7] van Griethuysen JJM, Fedorov A, Parmar C, Hosny A, Aucoin N, Narayan V, Beets-Tan RGH, Fillion-Robin JC, Pieper S, Aerts HJWL (2017). Computational Radiomics System to Decode the Radiographic phenotype. Cancer Res.

[8] Vujanić GM, Gessler M, Ooms AHAG, Collini P, Coulomb-l’Hermine A, D’Hooghe E, de Krijger RR, Perotti D, Pritchard-Jones K, Vokuhl C, van den Heuvel-Eibrink MM, Graf N (2018). International Society of Paediatric Oncology–Renal Tumour Study Group (SIOP–RTSG). The UMBRELLA SIOP-RTSG 2016 Wilms tumour pathology and molecular biology protocol. Nat Rev Urol.

[9] van den Heuvel-Eibrink MM, Hol JA, Pritchard-Jones K (2017). Position paper: Rationale for the treatment of Wilms tumour in the UMBRELLA SIOP-RTSG 2016 protocol. Nat Rev Urol.

[10] Provenzi VO, Rosa RF, Rosa RC, Roehe AV, dos Santos PP, Faulhaber FR, de Oliveira CA, Zen PR. Tamanho tumoral e prognóstico em pacientes portadores de tumor de Wilms [Tumor size and prognosis in patients with Wilms tumor]. Rev Paul Pediatr. 2015 Jan-Mar;33(1):82–7. 10.1016/j.rpped.2014.05.003. 10.1016/j.rpped.2014.05.003 PMC4436960 25623730

[11] JJoseph LL, Boddu D, Srinivasan HN, Regi SS, Antonisamy B, John R, Mathew LG, Totadri S (2022). Postchemotherapy tumor volume as a prognostic indicator in Wilms tumor: a single-center experience from South India. Pediatr Blood Cancer.

[12] Jiang Y, Yang M, Wang S, Li X, Sun Y (2020). Emerging role of deep learning-based artificial intelligence in tumor pathology. Cancer Commun (Lond).

[13] Tran KA, Kondrashova O, Bradley A, Williams ED, Pearson JV, Waddell N (2021). Deep learning in cancer diagnosis, prognosis and treatment selection. Genome Med.

[14] Zheng X, He B, Hu Y, Ren M, Chen Z, Zhang Z, Ma J, Ouyang L, Chu H, Gao H, He W, Liu T, Li G (2022). Diagnostic accuracy of Deep Learning and Radiomics in Lung Cancer staging: a systematic review and Meta-analysis. Front Public Health.

[15] Shimizu H, Nakayama KI (2020). Artificial intelligence in oncology. Cancer Sci.

[16] Isensee F, Jaeger PF, Kohl SAA, Petersen J, Maier-Hein KH (2021). nnU-Net: a self-configuring method for deep learning-based biomedical image segmentation. Nat Methods.

[17] Bailly A, Blanc C, Francis É, Guillotin T, Jamal F, Wakim B, Roy P (2022). Effects of dataset size and interactions on the prediction performance of logistic regression and deep learning models. Comput Methods Programs Biomed.

[18] Thian YL, Ng DW, Hallinan JTPD, Jagmohan P, Sia SY, Mohamed JSA, Quek ST, Feng M (2022). Effect of Training Data volume on performance of Convolutional Neural Network Pneumothorax Classifiers. J Digit Imaging.

[19] Kattan MW (2011). Factors affecting the accuracy of prediction models limit the comparison of rival prediction models when applied to separate data sets. Eur Urol.

[20] Wellens LM, Meulstee J, van de Ven CP, van Terwisscha CEJ, Littooij AS, van den Heuvel-Eibrink MM, Fiocco M, Rios AC, Maal T, Wijnen MHWA (2019). Comparison of 3-Dimensional and augmented reality kidney models with conventional Imaging Data in the Preoperative Assessment of Children with Wilms Tumors. JAMA Netw Open.

[21] Chaussy Y, Vieille L, Lacroix E, Lenoir M, Marie F, Corbat L, Henriet J, Auber F (2020). 3D reconstruction of Wilms’ tumor and kidneys in children: variability, usefulness and constraints. J Pediatr Urol.

[22] Tirumani SH, Shinagare AB, O’Neill AC, Nishino M, Rosenthal MH, Ramaiya NH (2016). Accuracy and feasibility of estimated tumour volumetry in primary gastric gastrointestinal stromal tumours: validation using semiautomated technique in 127 patients. Eur Radiol.

[23] Buser MAD, van der Steeg AFW, Wijnen MHWA, Fitski M, van Tinteren H, van den Heuvel-Eibrink MM, Littooij AS, van der Velden BHM (2023). Radiologic versus segmentation measurements to Quantify Wilms Tumor volume on MRI in Pediatric patients. Cancers (Basel).

[24] Ma XH, Shu L, Jia X, Zhou HC, Liu TT, Liang JW, Ding YS, He M, Shu Q (2022). Machine learning-based CT Radiomics Method for identifying the stage of Wilms Tumor in Children. Front Pediatr.

[25] Müller S, Farag I, Weickert J, Braun Y, Lollert A, Dobberstein J, Hötker A, Graf N (2019). Benchmarking Wilms’ tumor in multisequence MRI data: why does current clinical practice fail? Which popular segmentation algorithms perform well?. J Med Imaging (Bellingham).

[26] Kim HJ, Kim W (2012). Method of tumor volume evaluation using magnetic resonance imaging for outcome prediction in cervical cancer treated with concurrent chemotherapy and radiotherapy. Radiat Oncol J.

